# Formation and Characterization of Nanoparticles from Avocado Peel Extract and Bovine Serum Albumin

**DOI:** 10.3390/molecules31203649

**Published:** 2026-10-13

**Authors:** Lino Sánchez-Segura, Laura Elizabeth Zavala-García, Juan Vázquez-Martínez, Emir Martínez-Gutiérrez

**Affiliations:** 1 Departamento de Ingeniería Genética, Centro de Investigación y de Estudios Avanzados del Instituto Politécnico Nacional-Unidad Irapuato, Km 9.6 Libramiento Norte Carretera Irapuato-León, Irapuato 36824, Guanajuato, Mexico; lino.sanchez@cinvestav.mx (L.S.-S.); elizavala1993@gmail.com (L.E.Z.-G.); 2 Departamento de Ingeniería Química, Tecnológico Nacional de México/ITS Irapuato, Carretera Irapuato-Silao Km 12.5, Irapuato 36821, Guanajuato, Mexico; juan.vm@irapuato.tecnm.mx; 3 Secretaría de Ciencia, Humanidades, Tecnología e Innovación (SECIHTI)-Centro de Investigación y de Estudios Avanzados del Instituto Politécnico Nacional-Unidad Irapuato, Km 9.6 Libramiento Norte Carretera Irapuato-León, Irapuato 36824, Guanajuato, Mexico

**Keywords:** avocado peel extract, BSA, nanoparticles, phenolic compounds

## Abstract

Avocado peel is a source of polyphenols, which are generally light-sensitive and have low long-term stability. Nanoencapsulation could overcome this limitation. The aim of this work was to formulate bovine serum albumin (BSA) nanoparticles containing avocado peel extract and to determine the effect of increased extract concentration on their characteristics. We characterized the nanoparticles using transmission electron microscopy (TEM), ζ-potential and Fourier transform infrared (FTIR) spectroscopy. The results showed that nanoparticle yield decreased by 17.1% when 2.5 mg of extract was added compared with empty nanoparticles. The smallest nanoparticles, obtained with 2.5 mg of extract, had an effective diameter of 258.7 nm and a circular shape. As the amount of extract increased, the nanoparticle shapes shifted from elliptical to irregular, suggesting that the extract and its concentration affected the nanostructure. The FTIR spectra showed perturbations induced by the interaction between the extract and albumin. Significant FTIR peaks were observed at 3290 cm^−1^ and 1644 cm^−1^, suggesting that the extract was successfully encapsulated within the BSA matrix.

## 1. Introduction

Worldwide, a large amount of organic waste comes from food production, distribution and consumption. This includes seeds and peels, as well as fruits discarded because of their appearance, size and maturity. Avocado is a fruit consumed around the world, and its production has increased in recent years, reaching a global production of 11,355,378.8 tons in 2024 [1]. The seeds and peels are generally discarded but can be used as raw materials to obtain value-added compounds. In this sense, avocado peel is a waste material from which extracts with a high total phenolic content can be obtained, along with various bioactive compounds such as epicatechin, quinic acid, hydroxycinnamic acids, and procyanidins [2,3,4]. Epicatechin exhibits antioxidant, anti-inflammatory, and anti-diabetic properties [5]; procyanidins have been associated with reduced DNA damage through the activation of Nrf2 [6]; and chlorogenic acid exhibits antioxidant, antibacterial and neuroprotective activities [7]. Thus, different phytochemicals in avocado peel have been associated with various biological activities of interest in functional foods, nutraceuticals and other products that positively affect health [8]. Consequently, recent studies have been carried out to explore the potential of avocado peel extract, such as the study by Tremocoldi et al. (2018) [9], which reported that Fuerte avocado peel extracts suppressed the release of the inflammatory mediators nitric oxide and TNF-α (a pro-inflammatory cytokine), suggesting anti-inflammatory activity. Ferreira et al. (2022) [10] observed that avocado peel ethanolic extracts incorporated into topical emulsions acted as antibacterial and antioxidant agents.

However, when phenolic compounds are extracted from plants, they are more susceptible to degradation by light, oxygen, and pH than when they are maintained in their natural matrix [11]. The stability of natural extracts in terms of total phenolic content decreases over time, as reported in [4]. Total phenolic content (TPC) has been linked to antioxidant activity [12,13], which is associated with various health benefits [14]; therefore, it is desirable for natural extracts, such as avocado peel extract, to remain stable for longer periods. An alternative is encapsulation, in which one or more polymers form a matrix that envelops the compounds of interest. This process protects the compounds, increases their stability, and controls their release [15]. Nanoencapsulation is an efficient, promising technique for protecting and releasing bioactive compounds by using food proteins to form nanostructures such as nanoparticles [16].

Nanoparticles are characterized by ordered structures with sizes between 10 and 1000 nm; they can be formed from inorganic or organic materials [17]. In contrast, the synthesis of nanoparticles containing avocado peel extract has rarely been studied. Nevertheless, the few reported studies are promising. Cerda-Opazo et al. (2021) [18] prepared nanoemulsions loaded with a procyanidin-rich extract obtained from avocado peels. They achieved high encapsulation efficiency and observed preferential cytotoxicity against cancer cells when the nanoformulation was used. Furthermore, Sahyon et al. (2023) [19] studied an avocado peel-loaded chitosan nanocombination, which showed superior anticancer potency compared with avocado peel extract alone. Therefore, it is desirable to explore different encapsulating materials and their interactions with avocado peel extract to expand its potential applications. In this sense, bovine serum albumin (BSA) has been used to elaborate nanocarriers loaded with bioactive compounds [20]. BSA is a globular protein with several advantages, including biodegradability, low toxicity, low cost, capacity to encapsulate hydrophilic and hydrophobic molecules, accessible purification and nanoparticles that can be easily adapted to the human body [21,22]. Therefore, incorporating avocado peel extract into BSA nanoparticles represents a promising way to enhance stability while preserving its beneficial properties for a longer period. BSA’s applications stem from its versatile binding and capacity to load endogenous and exogenous molecules [23], such as natural products. However, this capacity decreases when BSA interacts with polyphenols and phenolic compounds due to strong reactions. In several reports on BSA nanoparticles loaded with phenolic compounds such as rutin, carnosic acid and epigallocatechin gallate, the drug load ranged from 0.6 to 10 mg to prevent the crosslinking effect of polyphenols [24,25,26]. However, the use of BSA as a matrix for encapsulating avocado peel extract as well as the effect of avocado peel extract concentration on nanoparticle formation have scarcely been evaluated. Thus, the present study aimed to obtain bovine serum albumin nanoparticles containing avocado peel extract and determine the effect of extract concentration on nanoparticle synthesis.

## 2. Results and Discussion

### 2.1. Total Phenolic Content in the Avocado Peel Extract

The total phenol content of avocado peel extract (Av-extract) was determined; for this purpose, 5 mg of Av-extract was diluted in 1 mL of deionized water, and a total phenolic content of 1055.7 mg GAE/L was obtained. However, when Av-extract was diluted in ethanol, the total phenol content (TPC) decreased by 50%. In this sense, Figueroa et al. (2021) [2] reported that the composition of solvent significantly affected the extraction of TPC and that when ethanol was used at 100% the TPC was lower.

### 2.2. Effect of the Concentration of Av-Extract on the Yield and Encapsulation of Total Polyphenols

Nanoparticle yield and the total polyphenolic content of the encapsulates are not parameters that describe how the molecules assemble to form nanostructures. However, they allow us to establish the capacity of the BSA molecules to entrap drugs and metabolites and the internal changes in the BSA that affect the supramolecular ensemble of nanoparticles. In the treatment with 0 mg of Av-extract, the BSA nanoparticle yield reached 93.0%, a higher yield than obtained in other works with a similar method of synthesis; for those NPs, the yields were around 60.0 and 72.4% [27,28] but similar to that reported by Bronze-Uhle et al. (2017) [29], indicating that BSA nanoparticles were successfully formed. When the extract was added to the formulation of nanoparticles (a low amount of 2.5 mg), the yield of the BSA decreased by 17.1% in comparison with empty nanoparticles (Table 1), and the quantity of total polyphenols was 94 mg GAE/L. These values indicate that polyphenols affect some amino acids during the solvation process, changing the capacity to entrap drugs. A subsequent treatment with 5.0 mg of Av-extract increased nanoparticle yield and total polyphenols to 82.03% and 192 mg GAE/L, respectively. In this condition, the encapsulation system reached the maximum polyphenol encapsulation. The maximal yield was achieved at 7.5 mg of Av-extract with a value of 102.45%. In comparison, total polyphenols decreased to 80 mg GAE/L, and the ratio between both parameters indicates that during assembly the albumin showed more affinity to other hydrophobic molecules found in the extract, for example, lipids such as octanoic and palmitic acid [3]. Finally, at 10 mg of Av-extract, the yield decreased by 35.2% compared with empty nanoparticles, and the quantity of total polyphenols was below the detection level; this effect was probably caused by the instability of the interaction of polyphenols and other molecules of the Av-extract with amino acids. In the case of polyphenols crosslinking activity with the amino acids has been reported, with a subsequent reduction in the transformation of BSA in nanoparticles [30]. Furthermore, different behaviors during the interaction of polyphenols and BSA have been observed that depend on the type of phenolic compound and other factors such as temperature, pH, salinity, and protein concentration that can impact the structure and stability of the protein [31]; for example, it has been reported that chlorogenic acid reduces the digestibility of protein [32]. Therefore, the components of Av-extract and their concentration could impact the physicochemical properties of nanoparticles.

### 2.3. Morphology of the BSA–Avocado Peel Extract Nanoparticles

The aspect of the nanoparticles changed according to the increase in avocado peel extract in the formulation. At 0 mg of Av-extract, the elliptical shape was the most observed morphology in the nanoparticles (Figure 1a); a similar morphology for empty nanoparticles was reported by Sánchez-Arreguin et al. (2019) [27]. At 2.5 mg of Av-extract, nanoparticles with circular shape and small size were observed (Figure 1b); this morphology at a low concentration of Av-extract (0.625 mg/mL) suggests that chemical compounds such as polyphenols were entrapped in the albumin. At 5.0 mg of Av-extract, the isolated nanoparticles showed an elongated shape (Figure 1c) like the elliptical shape observed at 0 mg Av-extract, though a possible explanation of this aspect is the coalescence between nanoparticles [27,33]. At 7.5 mg of Av-extract, the nanoparticles showed an elongated aspect due to the formations of clusters comprising between 6 and 10 nanoparticles (Figure 1d); this irregular aspect was probably due to coalescence between nanoparticles. At 10 mg of Av-extract, the nanoparticles did not lose their circular shape; however, their size increased, and they showed a coalescence, forming globular aggregates (Figure 1e). Indeed, it has been mentioned that a balanced ratio between drug and protein is crucial as it favors interactions and the cross-linking rate; an excess of drugs leads to increased particle size and coalescence [34]. This behavior could occur between the extract and the protein but in a more complex manner due to the number of compounds making up the extract and their proportions within it as these can interact with the protein in multiple ways. Proposed interactions between proteins and polyphenols (e.g., chlorogenic acid) include ionic interactions, hydrogen bonds, hydrophobic interactions, and covalent bonds, and interactions between proteins and flavonoids (such as epicatechin) include covalent and non-covalent interactions [35].

### 2.4. Morphometry of the BSA–Avocado Peel Extract Nanoparticles

Morphological analysis of nanoparticles established that their shape changes when the concentration of avocado peel extract increases. To clarify the magnitude of the shape change, the morphometric parameters were calculated from TEM images of the isolated nanoparticles. For the area (A) of the nanoparticles, this parameter decreased in the order of 2.3 times from 0 mg treatment to 2.5 mg treatment of Av-extract (Figure 2). However, in subsequent treatments, the A of the nanoparticles increased, reaching the highest level in the treatment with 7.5 mg of Av-extract, and showed a difference of size of 7.8 times with respect to the nanoparticles at 2.5 mg. At 10 mg of Av-extract, the area of the nanoparticles decreased, probably due to the saturation of the albumin molecules with the chemical compounds found in the extract. 

The shape factor (Sf) describes how similar an object is to a perfect circle [36]. The average of Sf was 0.43, 0.66, 0.36, 0.31 and 0.42 for 0, 2.5, 5.0, 7.5 and 10 mg Av-extract treatments, respectively (Figure 3).

The tendency is similar to A due to the Sf equation being calculated from this parameter. Therefore, nanoparticles with an approximately circular shape were found in treatments with low quantities of Av-extract. On the other hand, the aspect ratio (Ar) describes nanoparticle elongationand the 2.5 and 7.5 mg treatments showed similar values (Ar = 1.14 and 1.13, respectively) of this parameter. It was indicated that the nanoparticles showed symmetry between the axis that crossed the center of the circumference. The loss of symmetry was found in the treatment with 10 mg, with Sf = 1.57, indicating that the isolated nanoparticles are aggregates of several nanoparticles (Figure 4).

The effective diameter (Ed) is the most common parameter used to describe nanoparticle size. However, in this study, the diameter of the particles could be overestimated due to the aggregation. The Ed values were 434.9, 258.7, 450.2, 680.52 and 688.2 nm to 0, 2.5, 5.0, 7.5 and 10 mg, respectively (Figure 5). BSA–Av-extract nanoparticles with the lowest extract concentration (2.5 mg) showed the smallest size; they were even smaller than empty nanoparticles and had a circular shape. A similar behavior was observed in procyanidin-loaded nanoemulsions, which were smaller than blank nanoemulsions, suggesting that procyanidins (produced from avocado peel) had a structural effect in the formation of nanoemulsions due to their lipophilic characteristics and presented a spherical shape [18]. Thus, the procyanidins present in avocado peel extract could have a similar effect on nanoparticle formation at a lower Av-extract concentration, while at a higher concentration, the Ed increases. There exists a relation between the increasing concentration of the encapsulated compounds and nanoparticle size as well as the loss of shape of the nanoparticles; both results could be due to the critical instability of the molecules of BSA that form the scaffolding of the nanoparticle.

### 2.5. Surface Charge (ζ-Potential), Hydrodynamic Diameter, and Polydispersity Index (PDI)

The surface charge of the nanoparticles was affected by the concentration of Av-extract included in their formulation. In this study, the ζ-potential was more negative in treatments with low concentrations of Av-extract than in the treatment without extract. The values of ζ-potential were −46.7, −57.1, and −62.7 mV for 0, 2.5 and 5.0 mg, respectively (Figure 6). This tendency was observed in previous work in which capsaicin was encapsulated [33]. In this case, the increased negative charge probably resulted from polyphenols interacting with high-affinity amino acids on albumin, which then oriented these amino acids with their charged surfaces toward the nanoparticle to stabilize the nanostructure. Interestingly, in treatments with low concentration of Av-extract, the predominance of the isolated nanoparticles allowed the real charge of the nanoparticles to be measured. In contrast, at higher concentrations of 7.5 and 10 mg, a decrease in negative ζ-potential was observed with values of −38.3 and −44.5, respectively. This loss of negative surface charge may have resulted from nanoparticle coalescence and coagulation, leading to aggregation and loss of polyphenols in the nanostructure. In this sense, the addition of Av-extract increased the values of negative ζ-potential in treatments at 2.5 and 5.0 mg, promoting the stability of nanoparticles. Due to a higher positive or negative ζ-potential, the particle charge tends to repel, and, therefore, a minor tendency to aggregate could be observed [37]. However, in the two higher concentrations (7.5 and 10 mg) ζ-potential was similar to that observed at 0 mg, suggesting a diminished stability under the conditions evaluated. In this sense, it has been suggested that a more positive ζ-potential is associated with a larger number of amino groups on the surface of nanoparticles [38].

The hydrodynamic diameter (HD) and PDI are measures of the stability of the nanoparticles in suspension, generally in water. At 0 mg of Av-extract, the HD was 373 ± 45 and PDI was 0.30 ± 0.20; at 2.5 mg, the aggregated size decreased to 320 ± 20 and PDI 0.16 ± 0.07, indicating a monomodal distribution (i.e., homogeneous nanoparticle size), while at 5.0 mg of Av-extract, the HD was 372.15 ± 35.98 and PDI was 0.56 ± 0.27. The formation of coalescent nanoparticles is a possible cause of the distribution of the size, which showed a broad dispersion. The formation of aggregates showed a slight increase at 7.5 mg of Av-extract; the HD was 450 ± 60 and PDI was 0.6 ± 0.09, indicating that there were almost no single nanoparticles and that units of two or more nanoparticles formed. Similarly, at 10 mg of Av-extract, the nanoparticles showed greater instability, with precipitation and formation of the large aggregates. The HD showed a slightly decreased size (402 ± 60) and PDI was 0.69 ± 0.40, indicating that the aggregates are more compact and the distribution of the size and shapes is more diverse. In this sense, a PDI value between 0.1 and 0.25 has been associated with narrow size distribution, while a PDI greater than 0.5 has been associated with a broad distribution [39], which coincides with the results obtained in the present work. The results indicate that when the avocado peel extract was added at the lowest concentration, the interaction of the extract with the protein favored the formation of smaller and more narrowly distributed nanoparticles, but as the concentration of the extract was increased, the behavior was different.

### 2.6. Molecular Interaction of BSA with Av-Extract

To determine the functional groups present in native serum bovine albumin and Av-extract, the spectra were recorded and compared with those of previous reports. The solid line in Figure 7a corresponds to BSA and coincides with previous descriptions by Carriles et al. (2021) [28], Sánchez-Arreguin et al. (2019) [27] and Bronze-Uhle et al. (2017) [29]. In this work, the peak found at 1646 cm^−1^ acquired great importance due to the probable interaction with polyphenol compounds. This peak corresponds to a stretching of the carbonyl group (C=O) of amide I. On the other hand, the dashed line in Figure 7a corresponds to the spectrum of the Av-extract; this spectrum is characterized by a broad curve with a maximal peak at 3290 cm^−1^, possibly corresponding to O-H stretching regions of the phenolic compounds. However, due to the deformation of the peak, it is possible that other functional groups with protons, such as NH_2_, contributed to the signal. The shape of this peak is like the spectrum of the avocado peel and leaf; however, the value of the maximal peak is like the spectrum of avocado pulp (3284 cm^−1^) [40]. The peak at 1743 cm^−1^ was associated with the C=O stretching vibration (carbonyl functional group), which probably corresponds to aldehydes and ketones from triglycerides and polyphenols, respectively [28]. A minor peak was found at 1602 cm^−1^ corresponding to stretching cis C=C/C=O carbonyl, strongly associated with avocado pulp [40].

The NPs of BSA–Av-extract at 0, 2.5, 5.0, 7.5 and 10 mg showed changes in the peak that correspond to amide A of the native BSA (Figure 7b). BSA nanostructures showed a strong peak that reached the region from 3680 to 3104 cm^−1^, with maximal transmittance at 3288 cm^−1^ in BSA nanoparticles without extract (0 mg). Moreover, in the BSA–Av-extract nanoparticles, the interaction between BSA and the phenolic compounds caused changes in the peak position, with maximal transmittance at 3290 cm^−1^, and in the intensity of this peak with respect to the empty nanoparticles (0 mg), which suggests an interaction between polyphenols and free amine groups of BSA [41]. However, when the highest concentration of Av-extract (10 mg) was used, the intensity of the peak was like treatment with 0 mg, indicating a decrease in the Av-extract encapsulated, which coincides with the decrease observed in the yield BSA of the same treatment (Table 1). This effect in which the changes in the intensity of peak do not respond to the increase in Av-extract is probably due to aleatory interactions between the OH phenolic with NH_2_ of the amino acids of BSA.

In the NP spectra, a stronger peak was observed in the region from 1754 to 1587 cm^−1^, with maximum intensity at 1644 cm^−1^, in all treatments (Figure 7b). This change probably corresponds to the overlap between the carbonyl signal of polyphenols (1743 cm^−1^) and the amide I band of albumins (1646 cm^−1^) of the functional group. The gradual increase in spectral intensity from empty NPs (0 mg) to higher concentrations (10 mg) suggests stronger interaction of polyphenols (such as epicatechin) with the C=O and -NH groups of amino acids, modifying the arrangement of polypeptide carbonyl hydrogen-bonding patterns. This suggests the Av-extract was successfully encapsulated in the BSA matrix.

### 2.7. Quantification of Epicatechin and Chlorogenic Acid

HPLC quantification of epicatechin and chlorogenic acid in the extract yielded 6.76 and 51.01 mg/g of extract, respectively. However, the methodology used could not detect their presence in the nanoparticles. In this sense, there is evidence that indicates that the digestibility of BSA is diminished by conjugation with chlorogenic acid, exhibiting only partial digestion after of 24 h exposure to trypsin [42]. This may have affected the determination of the phenolic compounds. Furthermore, several factors can slow the release of compounds from the plant extract from nanostructures, including strong chemical interaction between the plant extract and the matrix and stiffening of the polymer network during crossslinking in the nanostructuring process [43]. Additionally, other factors could affect compound recovery, such as losses during drying or washing or incomplete extraction.

### 2.8. Spectral Fluorescence Fingerprint of Nanoparticles

Some plant components showed autofluorescence, mostly attributed to light-absorbing pigments and secondary metabolites that absorb photons in several regions of UV light and emit fluorescence in the visible region of the spectral wavelength [44]. To obtain the Av-extract fingerprint, scanned excitation wavelength from 695 nm to 1000 nm using multiphoton microscopy, and we found that the avocado peel extract fluoresced at 700 nm with 2.5% laser power. The raw Av-extract showed a stronger peak emission at 665 nm, with a higher fluorescence intensity (black solid line in Figure 8a), while in empty NPs (0 mg), the spectral signal of the polyphenols was not found (black dotted line in Figure 8a).

Interestingly, nanoparticles loaded with different concentrations of Av-extract showed fluorescence when excited at 700 nm. At 5.0 mg of Av-extract, the nanoparticles showed the highest fluorescence intensity with two peaks: the first peak was found at 660 nm and a second peak with minor intensity at 650 nm. Subsequently, the intensity of fluorescence decreased in the order of 7.5, 2.5 and 10 mg of Av-extract; although this last treatment involved a large quantity of extract, it is not correlated with the spectral intensity.

On the other hand, the BSA spectral fingerprint was found at 900 nm wavelength excitation, with 2.5% laser power. The spectral fingerprint of the empty NPs (0 mg) showed a maximal emission peak at 525 nm (Figure 8b, light gray solid line); this spectrum is considered the baseline of the nanostructured BSA. In contrast, in raw Av-extract, the BSA spectrum was not found (Figure 8b, black dotted line). The treatment with 2.5 mg of Av-extract showed major intensity emission with respect to the treatment with 0 mg; similarly the maximal peak emission was at 525 nm (Figure 8b, light gray solid line). Subsequently, the intensity emission decreased in treatments at 10, 5.0 and 7.5 mg, while maximal peak emission was 535, 520 and 535 nm. These changes in the maximal peak emission and the increase in the BSA signal were probably due to the interaction of the BSA with the polyphenols of the Av-extract.

Our physicochemical characterization shows that BSA nanoparticles loaded with Av-extract at low concentrations (2.5 and 5.0 mg) displayed optimal properties suitable for administration to cell cultures. However, nanoparticles with higher Av-extract concentrations (7.5 and 10 mg) showed adverse effects, such as coalescence and morphometric parameters indicating instability and aggregate formation. Notwithstanding, instability at higher extract concentrations is probably attributed to two factors, namely, the stronger interaction of phenolic compounds with BSA, which affects the physicochemical properties of proteins such as solubility and stability [31], and the interaction of glutaraldehyde with the Av-extract, which changes the interaction with the BSA and produces significant changes in PDI, ζ-potential [45], nanoparticle size [46], and coalescence [47]. This probable effect contrasts with the traditional description of the glutaraldehyde effect in nanoparticle formulations. Langer et al. (2003) [48] and Rahimnejad et al. (2012) [49] claim that glutaraldehyde concentrations did not affect particle size or coalescence. Another potential risk of glutaraldehyde is cytotoxicity from residual glutaraldehyde retained during crosslinking. Gough et al. (2002) [50] reported that residual glutaraldehyde toxicity caused apoptosis in cultured osteoblasts. A similar effect was observed in human vascular endothelial cell (HUVEC) cultures exposed to human and bovine serum albumin nanoparticles; cell viability decreased after 96 h as the concentration of glutaraldehyde in the particle formulation increased [51]. In both cases, glutaraldehyde remains part of the nanoparticles, increasing the risk of glutaraldehyde exposure. Therefore, it would be advisable in future studies to replace glutaraldehyde with a non-toxic crosslinking agent; it would even be possible to evaluate the formation de BSA– Av-extract without the addition of a crosslinking agent.

On the other hand, quantifying the phenolic compounds loaded in the nanoparticles by chromatography requires separating natural products from the BSA matrix. Chemical disassembly of BSA and physical disruption by sonication failed to separate both components. However, we confirmed the interaction between BSA and the Av-extract by FTIR, and microspectroscopy suggests that the phenolic compounds of the Av-extract were probably incorporated into the amino acid structure, forming a complex of amino-phenolics. Liu et al. (2023) [52] found, by molecular docking, that proanthocyanidins, catechin, epigallocatechin gallate, and tea polyphenols bound to BSA to form a polyphenol–BSA complex. Some polyphenols and phenols, such as catechin and epicatechin, interact with Trp134, Trp214 and Trp213 in BSA and HSA [53]. There probably exists a synergistic effect with the crosslinking mechanisms of glutaraldehyde, in which two carbonyl groups attack the amino groups of lysine and arginine residues in the guanidino side chains of BSA, forming Schiff bases via a condensation reaction [45,54]. In our BSA–Av-extract formulation, adding polyphenols makes BSA more compact, promoting coalescence and precipitation, which improves during the nanostructuring process at low concentrations of Av-extract and disrupts the ensemble process at high concentrations of Av-extract. Our work suggests that, in a proper formulation, phenolic compounds may serve a dual role as antioxidant agents and crosslinking agents with BSA. This enables some phenolic compounds to serve as substitutes for glutaraldehyde in albumin nanoparticle formulations.

## 3. Materials and Methods

### 3.1. Chemicals

The biological reagent used in this study was BSA (lyophilized powder, 66 kD) (Equitech-Bio, Kerrville, TX, USA). The chemical reagents were glutaraldehyde 25% (Electron Microscopy Science, Hatfield, PA, USA), sodium chloride analytical grade (Merck, Darmstandt, Germany), and absolute ethanol (≥99.8%) (Merck, Darmstandt, Germany). Folin–Ciocalteu phenol reagent and gallic acid were purchased from Sigma-Aldrich. All other reagents were analytical-grade.

### 3.2. Avocado Peel Extract

The avocado peel (var Hass) was collected from the residual material from local restaurants and homes. Avocado peel extract was obtained as described by Martínez-Gutiérrez (2023) [3]; first, the peels were rinsed, cut, ground (40 g plus 200 mL water) and subsequently macerated with agitation for 1 h at 70 °C. Lastly, the extract was lyophilized in Freezemobile 12 (Virtis) and was shielded from light until use.

### 3.3. Preparation of BSA–Av-Extract Nanoparticles

Nanoparticles were synthesized by the desolvation technique as reported by Langer et al. (2003) [48]; the protocol was modified by Sánchez-Arreguin et al. (2019) [27]. Five assays involving different amounts of lyophilized avocado peel extract (0, 2.5, 5.0, 7.5, or 10 mg) were carried out in triplicate. First, BSA powder (200 mg) was dissolved in 2 mL of 1 mM NaCl solution, pH 9.4 (±0.2). The solution was maintained with agitation at 200 rpm using a stirrer (Eurostar 20, IKA, Wilmington, NC, USA) for 30 min at room temperature. Prior to addition, the lyophilized extract was adjusted according to the approach of Pedrozo et al. (2020) [24], Khella et al. (2022) [25] and Martano et al. (2025) [26] and dissolved in 4 mL of ethanol, and the following concentrations were obtained: 0, 0.625, 1.25, 1.875 and 2.5 mg of extract/mL. Subsequently, the extract was sonicated (Ultrasonic equipment, PS-20A, Shenzhen Jie Tai Co., Ltd., Guangdong, China) at 40 KHz and 40 °C for 25 min and filtered through a 0.45 μm syringe filter (Sartorius, Goettingen, Germany). The solution extract was added to a rate of 1.0 mL/min with agitation at 600 rpm stirring speed. Lastly, chemical crosslinking was realized by adding 5 mL of 4% glutaraldehyde in a 1 mM NaCl solution, maintaining the agitation at 600 rpm for 30 min in dark conditions. The BSA nanoparticles loaded with avocado peel extract (BSA–Av-extract nanoparticles) and BSA nanoparticles were stored at 4 °C in dark conditions until their use.

### 3.4. Quantification of Nanoparticle Conversion

Volumes of 1 mL of nanoparticles were centrifuged at 12,485× *g* for 10 min (MC-12V, DuPont, Newtown, CT, USA); the supernatant was discarded and the pellet was resuspended in 1 mL of deionized water, then centrifuged at 12,485× *g* for 10 min at room temperature. This process was repeated 3 times. The pellet was dispersed by stirring with a vortex mixer (MX-S, SCILOGEX, Rocky Hill, CT, USA) for 5 min, then it was lyophilized (HETO MAXY dry, LYO, Lillerød, Denmark) and weighed. For the estimation of the BSA nanoparticle yield, a total volume of 11 mL was considered. The yield was estimated using Equation (1).

(1) $$\mathrm{Nanoparticles yield}\left(\%\right)=\frac{\mathrm{BSA in nanoparticle}(\mathrm{mg})}{\mathrm{Initial BSA}(\mathrm{mg})}\times 100$$

### 3.5. Quantification of Encapsulated Polyphenols

The Folin–Ciocalteu method was used to evaluate total phenolic content according to Singleton and Rossi (1965) [55] with modifications. A volume of 100 µL of nanoparticles (previously diluted at 1:5 with deionized water) was mixed with 500 µL of Folin–Ciocalteu reagent (0.2 N) and 400 µL of Na_2_CO_3_ (7.5%). The samples were homogenized and incubated for 30 min in darkness at room temperature. The samples were measured by absorption at 760 nm using an LKB spectrophotometer (Ultrospec III Pharmacia LKB, Chesapeake, VA, USA). The total phenolic content (TPC) was calculated from a standard curve of gallic acid; the results are expressed as gallic acid equivalents per L of the supernatant or avocado peel extract.

Additionally, the quantification of epicatechin and chlorogenic acid was carried out by HPLC using an Agilent 1200 series chromatograph (Agilent Technologies, Waldbronn, Germany) equipped with a UV-Vis detector with a diode array using a YMC-ODS-AM C-18 column (250 mm × 4.6 mm). Previously, 100 µL of nanoparticle suspension plus 900 µL of acetonitrile were mixed and sonicated for 30 min; the supernatant was recovered by centrifugation. After 100 µL of the sample was automatically injected into the chromatograph, the composition of the mobile phase was as follows: acetic acid (1%) in water (phase A) and (100%) acetonitrile (phase B) with a gradient elution: 0 min, 0% B; 5 min, 5% B; 10 min, 10% B; 30 min 15% B; 40 min, 15% B; 42 min, 0% B. The flow was 1 mL/min with λ = 280 nm at 25 °C.

### 3.6. Fourier Transform Infrared (FTIR) Spectroscopy

In FTIR analysis, samples of avocado extract and BSA (standard) were analyzed directly, whereas nanoparticles were previously washed with deionized water by three cycles of centrifugation 12,485× *g* for 8 min (MC-12V, DuPont, Newtown, CT, USA). The precipitate was dried using a freeze-dryer (HETO MAXY dry, LYO, Lillerød, Denmark)) for 3 h and then homogenized and analyzed. Infrared spectra were recorded using an FTIR spectrometer (Cary 660, Agilent Technologies, Santa Clara, CA, USA) with an attenuated total reflectance (ATR) fit and spectral range of 650–4000 cm^−1^. Spectral data were the result of sixty-four scans measured in mode transmittance, with a nominal resolution of 4 cm^−1^. Three independent replicates of each sample were averaged per spectrum. Spectra were acquired using SpectraGryph software version 1.2 (Dr. Friedrich Menges Software-Entwicklung), and the curve fitting was analyzed by SigmaPlot 12 (Systat Software, Inc., Sausalito, CA, USA).

### 3.7. Determination of ζ-Potential, PDI and Hydrodynamic Diameter of Aggregates

The ζ-potential, PDI and hydrodynamic diameter were realized using a Zetasizer Nano ZSP (Zetasizer Nanoanalyser ZS-90P, Malvern Instruments, Malvern, UK), using a folded capillary ζ cell (DTS1070, (Zetasizer Nanoanalyser ZS-90P, Malvern Instruments, Malvern, UK) and a forward scatter angle of 12.8°. The samples were diluted in a proportion of 1:20 using deionized water as solvent, to improve the measurement [27]. The values of ζ-potential were calculated using the Smoluchowski equation [56].

### 3.8. Transmission Electron Microscopy (TEM) and Morphometric Analysis of Nanoparticles

The morphology characterization of nanoparticles was realized by a transmission electron microscope Morgagni M-268, Philips/FEI, Brno, Czech Republic). For this purpose, 7.5 μL of nanoparticles were placed onto a 200-mesh copper grid coated with formvar/carbon (Ted Pella Inc., Redding, CA, USA) and incubated for 7 min. Later, the nanoparticles were contrasted with 2.0% uranyl acetate (Electron Microscopy Science Inc., Hatfield, PA, USA) and incubated for 7 min plus. The microscope was operated at 80 kV high voltage (EHT), vacuum pressure of 5 × 10^−3^ Pa, between 8900× and 9000× for low magnification and between 56,000× and 180,000× for high magnification. Micrographs were captured in.tif format (1376 × 1032 pixels) in grayscale. The zero was assigned to black and 255 to white in this scale. The parameters studied were area (A), shape factor (Sf), effective diameter (Ed), and aspect ratio (Ar), which was calculated according to Syverud et al. (2007) [57]. The A was quantified as the total number of pixels that an object occupies in the full image [58]. The pixel count can be calibrated to metric dimensions. Ar describes the relation between the width and length of the particles and was determined according to Parakhonskiy, (2015) [59], and Sf or circularity was calculated according to Bouwman et al. (2004) [36].

### 3.9. Microspectroscopy by Multiphoton Microscopy

Microspectroscopy was conducted to confirm the entrapped polyphenol compounds from avocado peel extract in BSA nanoparticles. The BSA–Av-extract nanoparticles were examined by the microspectroscopy technique according to Sánchez-Arreguin et al. [17]. The avocado extract and nanoparticles were mounted in glass slides and dried at 37 °C for 30 min in darkness. Subsequently, the samples were embedded with anti-fade fluorescence mounting medium (Abcam, Cambridge, UK) and covered with high-performance Zeiss cover glasses (D = 0.17 ± 0.005 mm, refractive index = 1.5255 ± 0.0015, Abbe number = 56 ± 2). Detection of BSA and polyphenols was performed by a multiphoton microscopy system (LSM 880-NLO, Zeiss, Oberkochen, Germany) coupled to a Ti:Sapphire infrared laser (Chameleon vision II, COHERENT, Santa Clara, CA, USA) with tuning capability in the range of 690–1060 nm. Analyses were performed using an immersion objective (63×/1.4, NA ∞–0.17, Zeiss Plan NEOFLUAR). The polyphenol compound spectrum and BSA were determined with a chameleon laser scanned at 695, 700, 750, 800, 850, 900, 950 and 1000 nm with 3.0% power and detected from 400 to 675 nm with a GaSP photomultiplier at 900 mV of sensibility. Lambda files were captured in CZI format at 1131 × 1131 pixels and RGB color. Spectral graphs were performed in Sigma plot 12 (Systat Software, Inc., Sausalito, CA, USA).

## 4. Conclusions

Analysis of the characterization parameters of BSA–avocado peel extract nanoparticles showed that the extract concentration affects nanoparticle formation and nanostructure. At 2.5 mg of Av-extract the nanoparticles had a circular shape and the smallest size. However, when the extract amount increased to 5 mg or more, the size increased, and the shape changed from elliptical to an irregular shape due to the coalescence between nanoparticles. Thus, the shape and size of the BSA–avocado peel extract nanoparticles were dependent on the BSA/avocado peel extract ratio, while the ζ-potential was more negative in the nanoparticles formulated with 5.0 mg of extract, followed by the nanoparticles with 2.5 mg of extract. FTIR analyses of the nanoparticles showed variations attributable to interactions between BSA and avocado peel extract, suggesting that this was successfully encapsulated into BSA matrix. However, a limitation of the study is that complete recovery of the extract was not possible, which restricted the performance of biological activity tests.

## Figures and Tables

**Figure 1:**
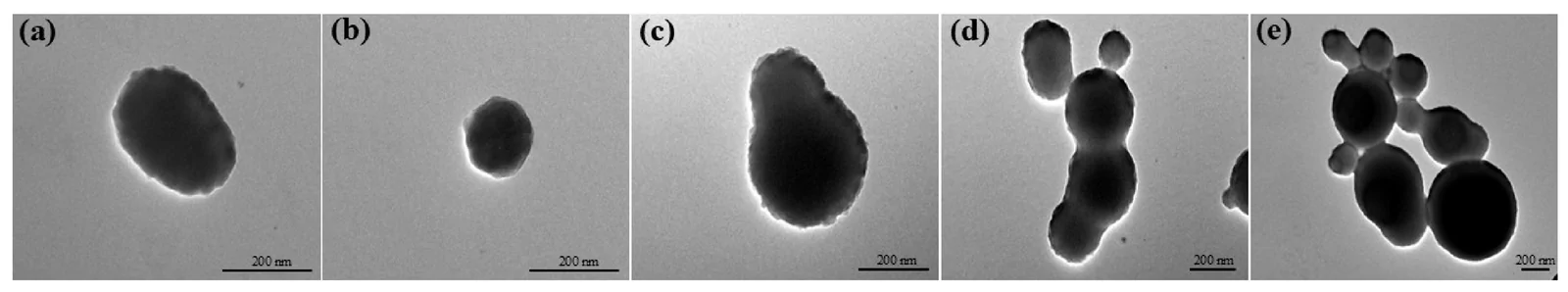
Morphology of the isolated nanoparticles acquired by TEM of the different amounts of avocado peel extract: (**a**) 0 mg of Av-extract, (140,000×) (**b**) 2.5 mg of Av-extract, (140,000×) (**c**) 5.0 mg of Av-extract, (110,000×) (**d**) 7.5 mg of Av-extract (71,000×) and (**e**) 10 mg of Av-extract (44,000×).

**Figure 2:**
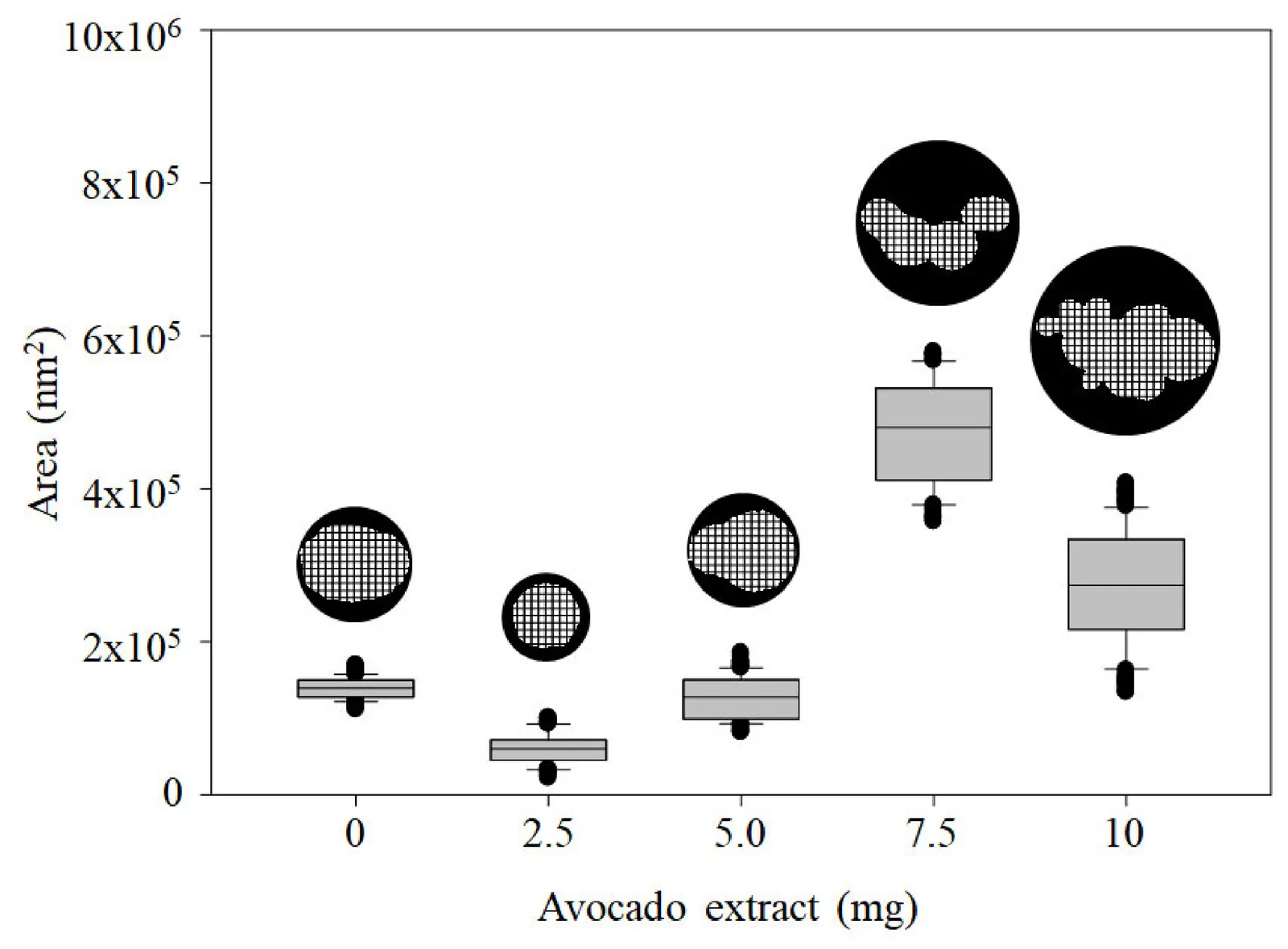
The morphometric parameter of Area (A) of isolated nanoparticles loaded with avocado peel extract. The A of nanoparticles decreased at a low concentration of Av-extract while increasing at higher amounts of avocado peel extract. The values are means of three experimental replicates ± standard deviations.

**Figure 3:**
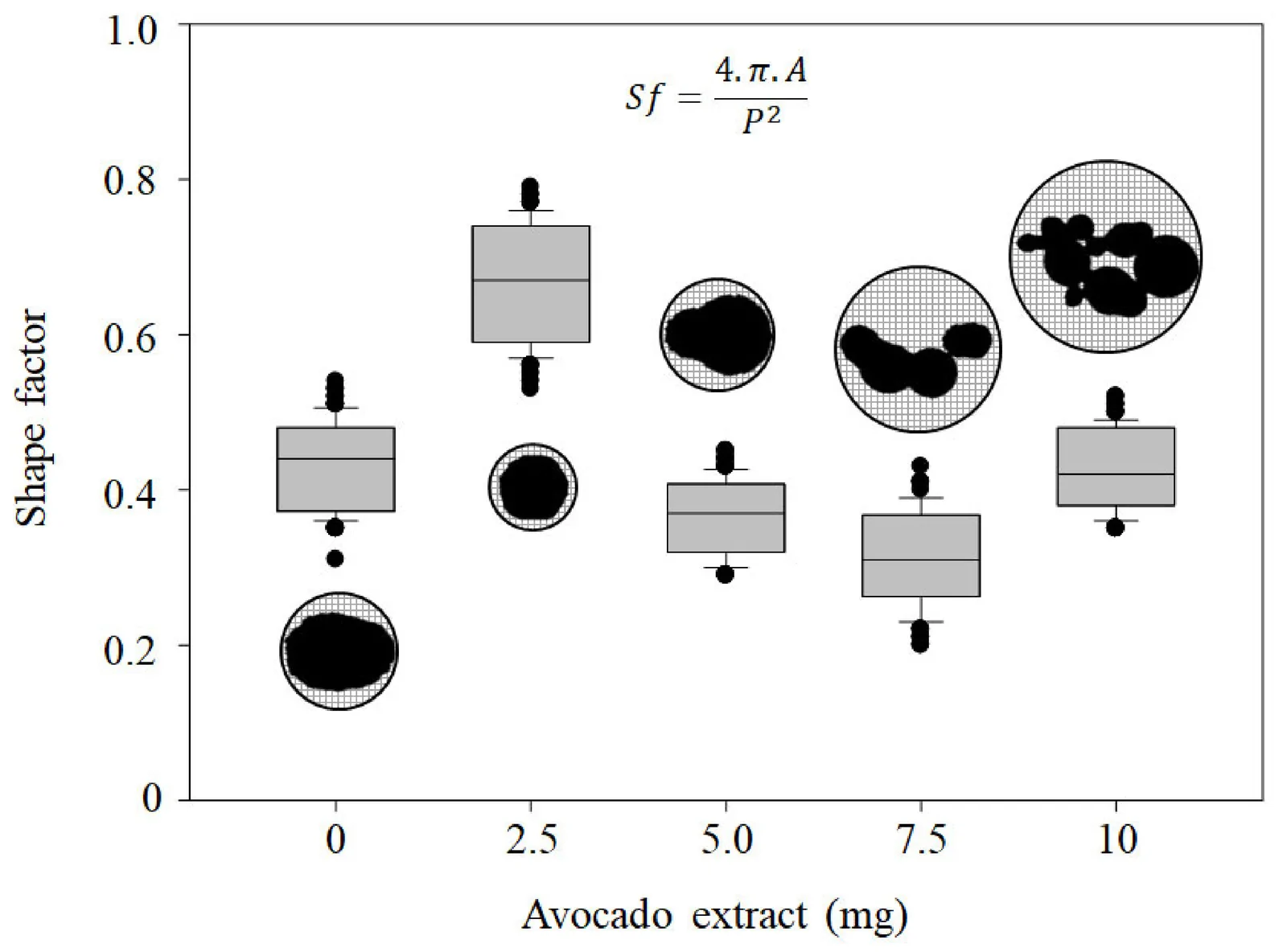
The morphometric parameter of shape factor (Sf) of isolated nanoparticles loaded with avocado peel extract. The Sf increased according to the concentration of Av-extract. The values are means of three experimental replicates ± standard deviations.

**Figure 4:**
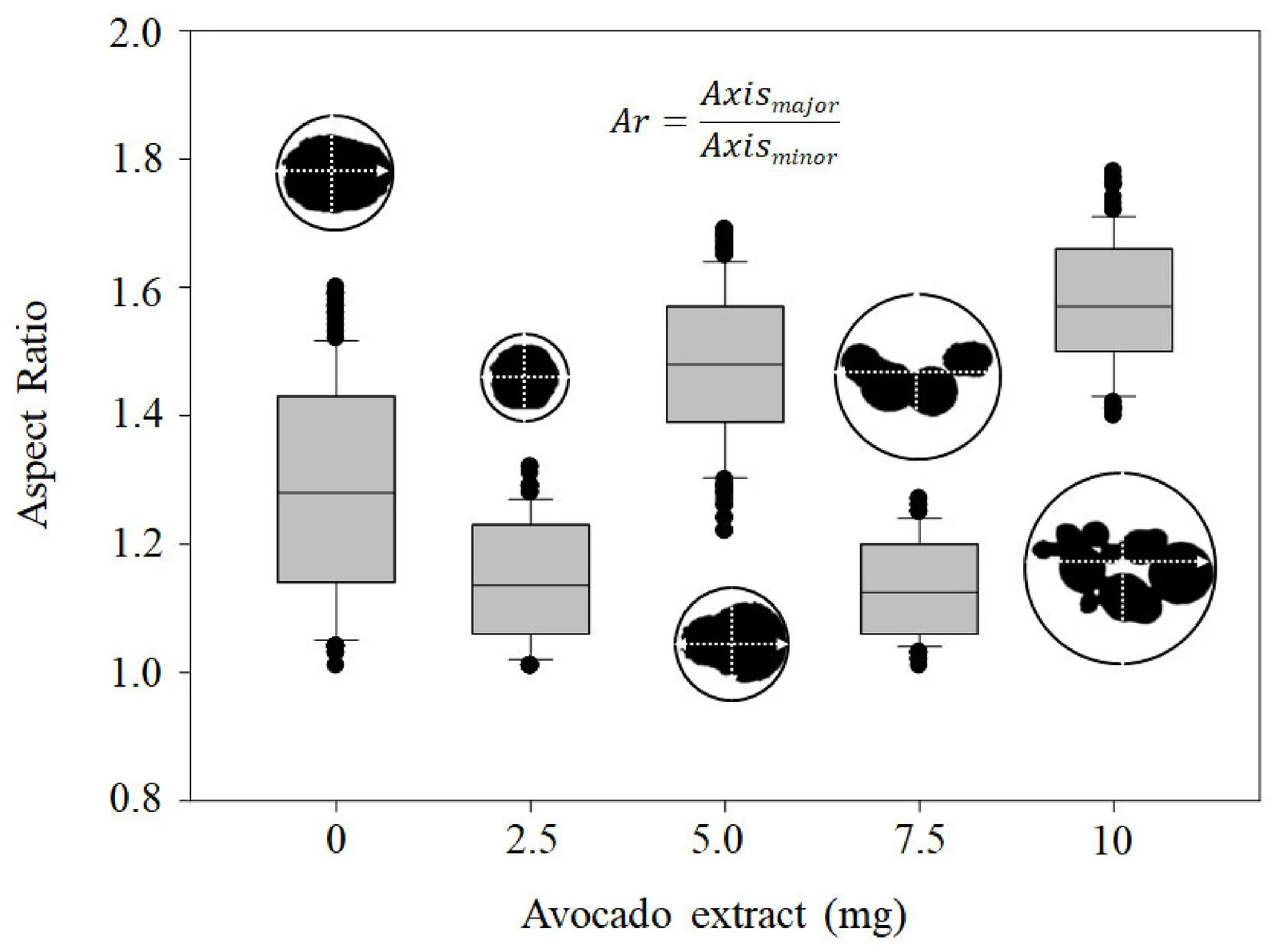
The morphometric parameter of aspect ratio (Ar) of isolated nanoparticles loaded with avocado peel extract. The Ar parameter fluctuated (decreased and increased) due to the entrapped Av-extract. The values are means of three experimental replicates ± standard deviations.

**Figure 5:**
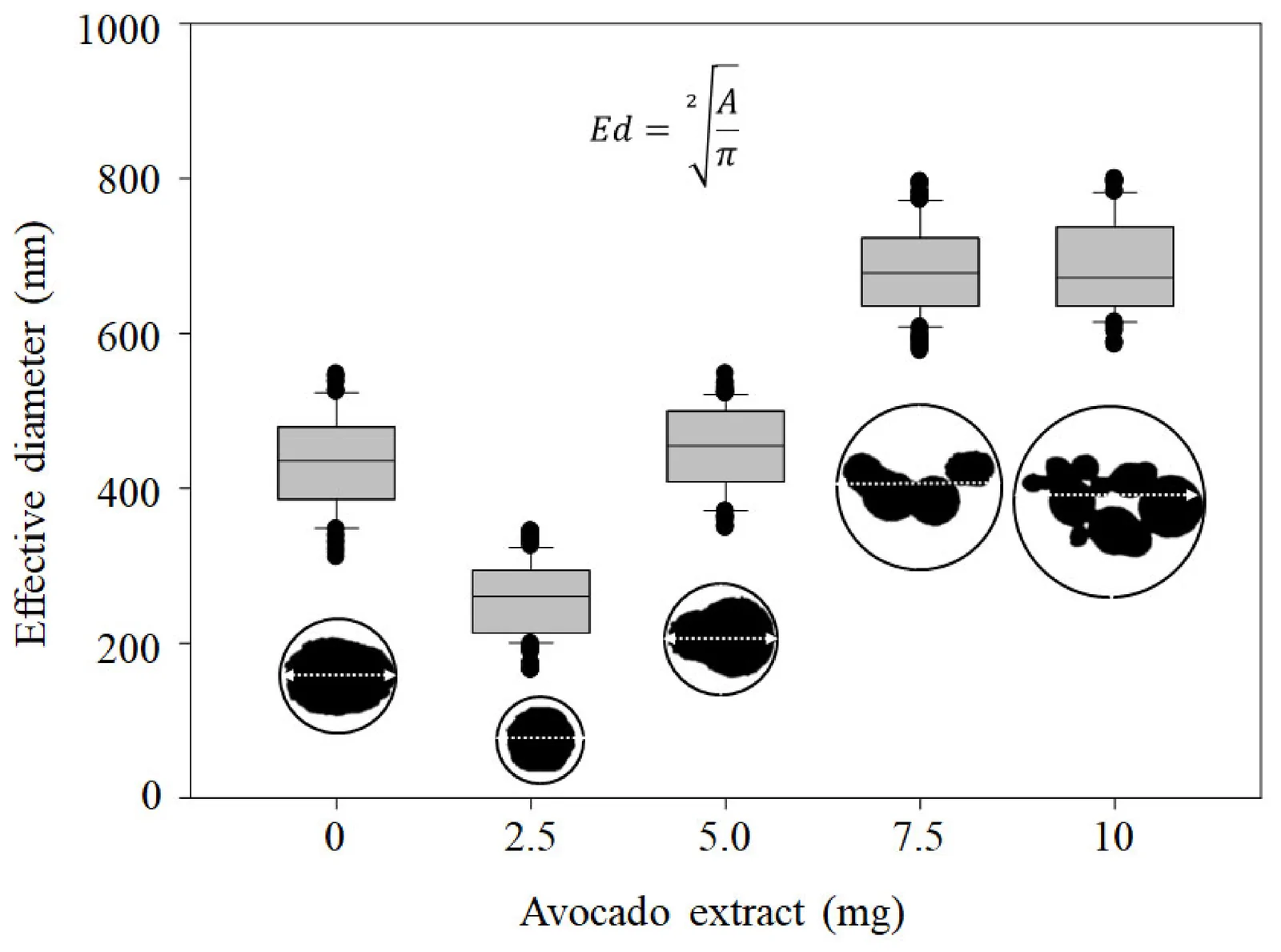
The morphometric parameter of effective diameter (Ed) of isolated nanoparticles loaded with avocado peel extract. The Ed parameter decreased at a low concentration of Av-extract (2.5 mg). In contrast, it increased at a higher concentration of Av-extract (7.5 and 10 mg). The values are means of three experimental replicates ± standard deviations.

**Figure 6:**
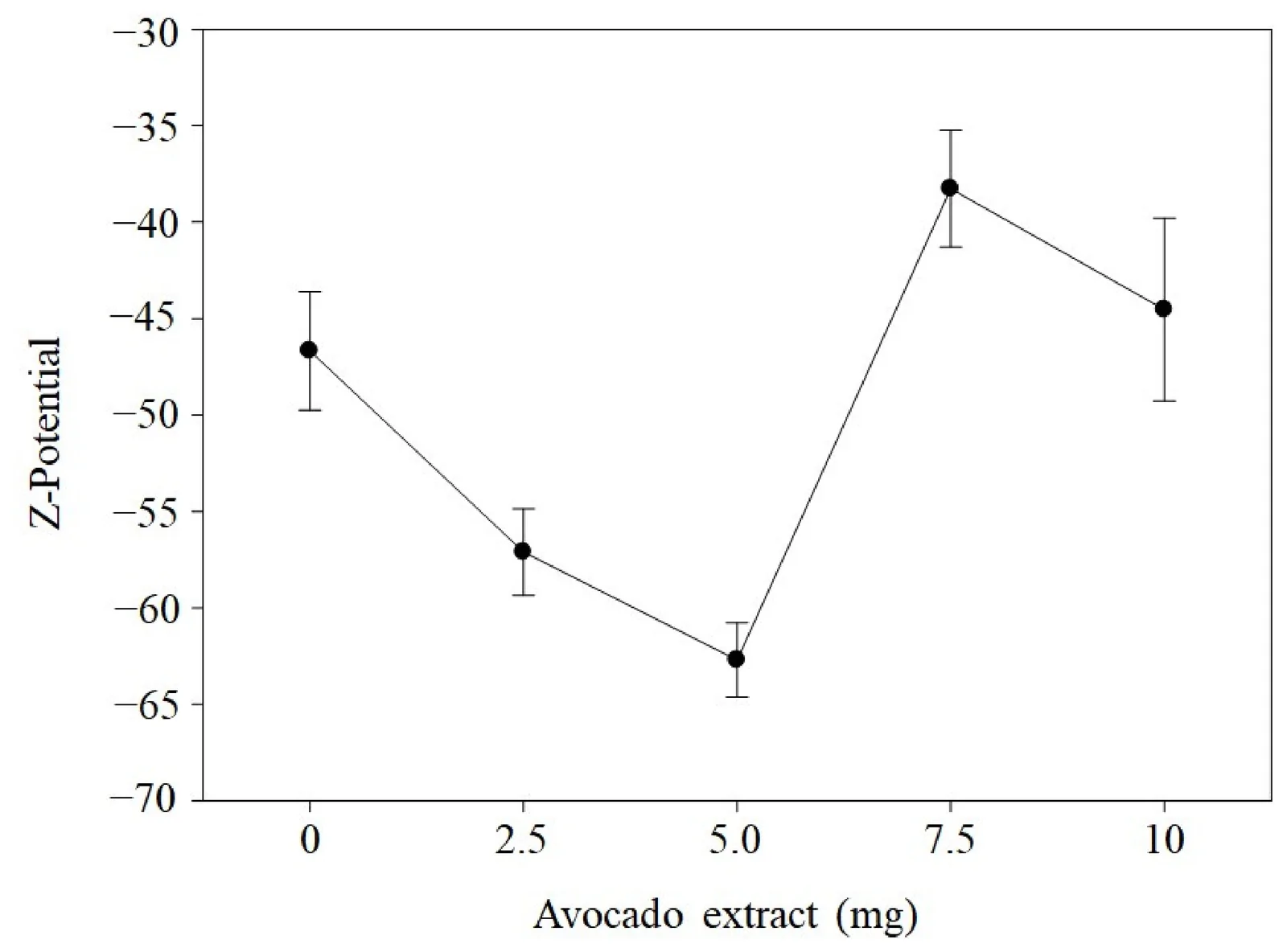
The ζ−potential of nanoparticles loaded with several concentrations of avocado peel extract. The ζ−potential showed a strong electronegativity at 5.0 mg of Av−extract, while the lower electronegativity was found at 7.5 mg. The values are means of three experimental replicates  ± standard deviations.

**Figure 7:**
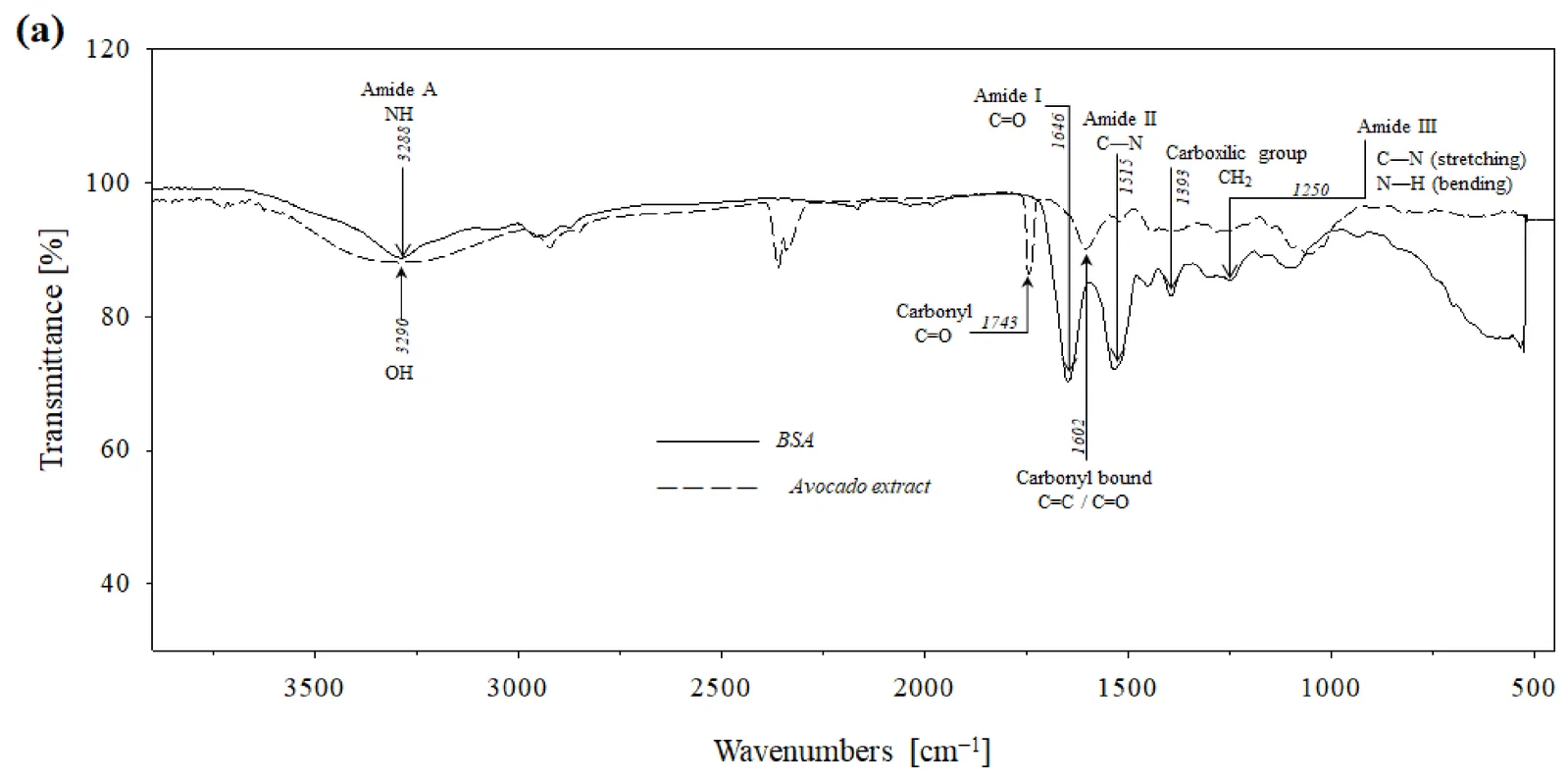

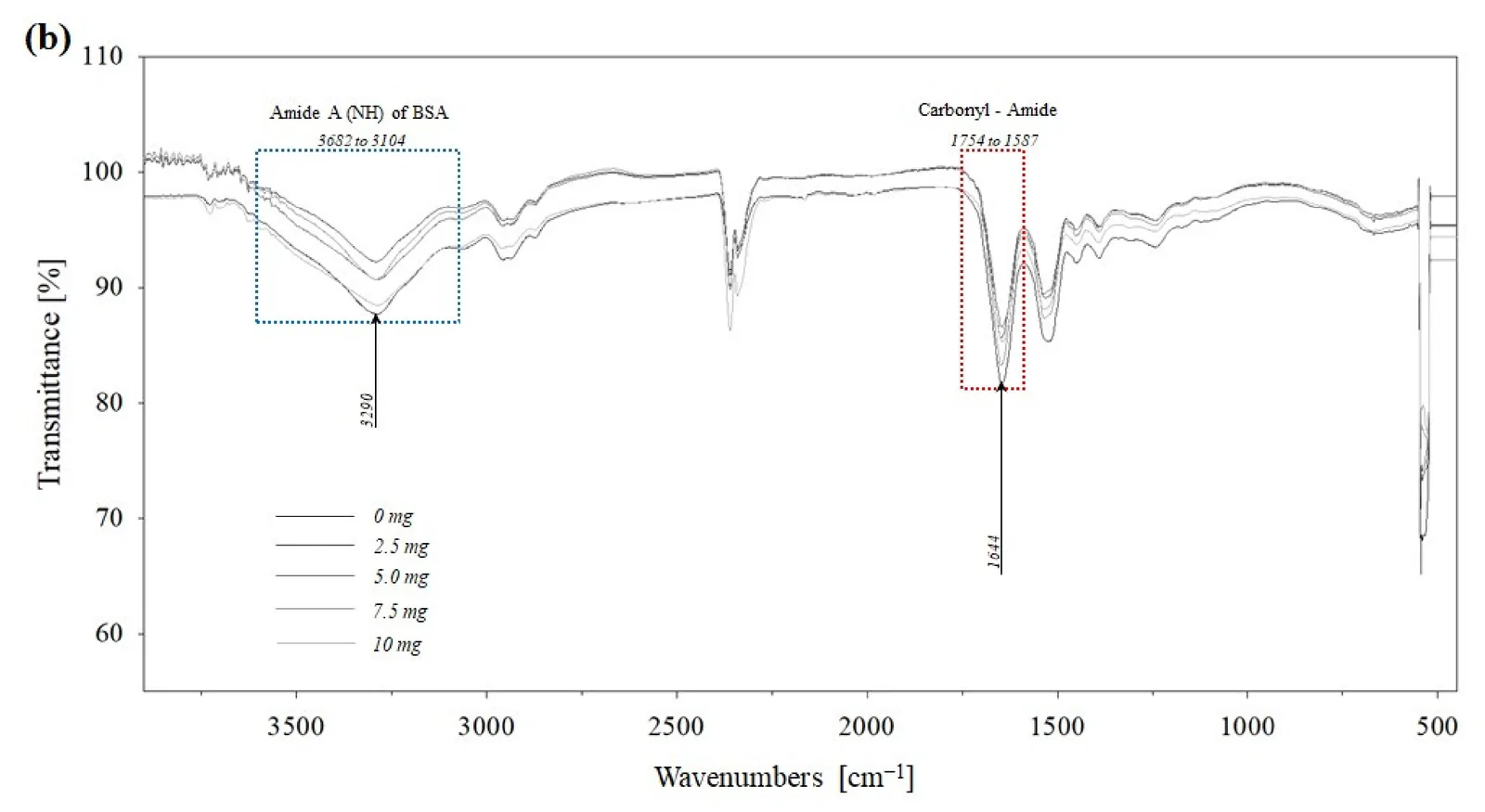
(**a**) FTIR spectra of raw avocado peel extract (dashed line) and the BSA protein standard (continuous line). (**b**) The FTIR spectra of different treatments of BSA−Av-extract nanoparticles. (a) 0 mg in black line; (b) 2.5 mg in dark gray line; (c) 5.0 mg in medium gray line; (d) 7.5 mg in light gray line and (e) 10 mg of Av−extract in bright gray line.

**Figure 8:**
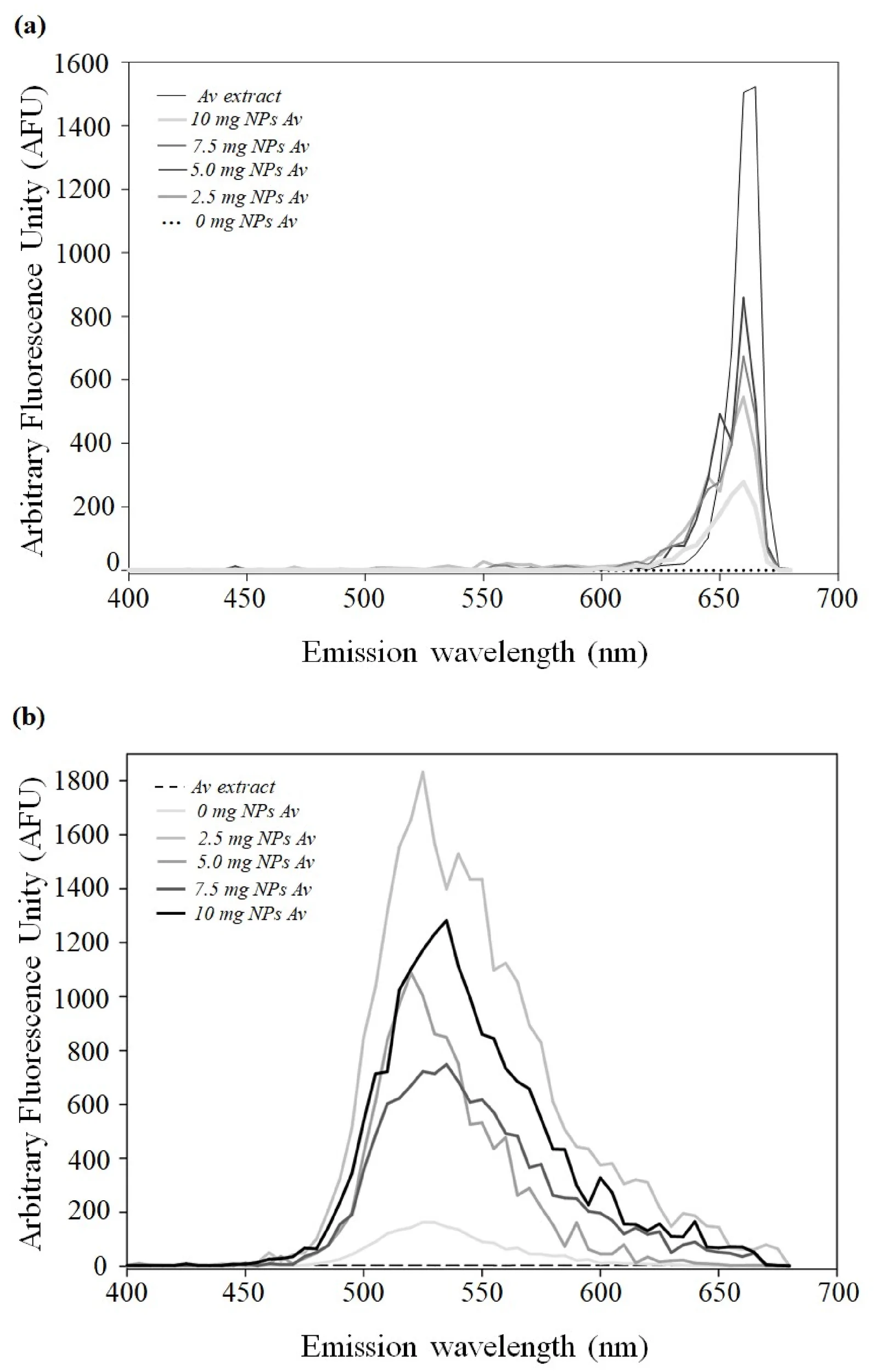
Microspectroscopy by multiphoton microscopy. (**a**) The peaks found at 700 nm with 2.5% laser power correspond to encapsulated polyphenols. (**b**) The peaks found at 900 nm with 2.5% laser power correspond to BSA of the nanoencapsulation matrix.

**Table 1:** Yield of empty BSA nanoparticles and BSA Av-extract nanoparticles.

Av-Extract Added (mg)	BSA Nanoparticle Yield
0	93.00 ± 1.91
2.5	77.11 ± 1.1
5.0	82.03 ± 1.04
7.5	102.45 ± 0.34
10.0	60.26 ± 8.35

Values are means of three experimental replicates.

## Data Availability

The original contributions presented in this study are included in the article. Further inquiries can be directed to the corresponding author.

## Abbreviations

The following abbreviations are used in this manuscript:

BSA	Bovine serum albumin
TEM	Transmission electron microscopy
FTIR	Fourier transform infrared
Av-extract	Avocado peel extract
GAE	Gallic acid equivalents
TPC	Total phenol content
NPs	Nanoparticles
A	Area
Sf	Shape factor
Ar	Aspect ratio
Ed	Effective diameter
PDI	Polydispersity Index
HD	Hydrodynamic diameter
HPLC	High-Performance Liquid Chromatography

## References

[1] Food and Agriculture of the United Nations Agriculture Database. https://www.fao.org/faostat/en/#data/QCL.

[2] Figueroa J.G., Borrás-Linares I., Del Pino-García R., Curiel J.A., Lozano-Sánchez J., Segura-Carretero A. (2021). Functional ingredient from avocado peel: Microwave-assisted extraction, characterization and potential applications for the food industry. Food Chem..

[3] Martínez-Gutiérrez E. (2023). Study of influence of extraction method on the recovery bioactive compounds from peel avocado. Molecules.

[4] Giannini G., Duque J.D.C., Molina-Hernandez J.B., Benavides W.R.V., Andrade-Mahecha M.M., Mejia J.F.G., Martinez-Correa H.A., Dalla Rosa S.T.M., Rocculi P. (2026). Sustainable Valorization of Avocado By-Products: Green Extraction of Phenolics with NaDES and Their Use in Fresh-Cut Fruit Preservation. Foods.

[5] Qu Z., Liu A., Li P., Liu C., Xiao W., Huang J., Liu Z., Zhang S. (2021). Advances in physiological functions and mechanisms of (−)-epicatechin. Crit. Rev. Food Sci. Nutr..

[6] Suraweera T.L., Merlin J.J., Dellaire G., Xu Z., Rupasinghe H.V. (2023). Genistein and procyanidin B2 reduce carcinogen-Induced reactive oxygen species and DNA damage through the activation of Nrf2/ARE cell signaling in bronchial epithelial cells in vitro. Int. J. Mol. Sci..

[7] Naveed M., Hejazi V., Abbas M., Kamboh A.A., Khan G.J., Shumzaid M., Ahmadg F., Babazadeh D., FangFang X., Modarresi-Ghazani F. (2018). Chlorogenic acid (CGA): A pharmacological review and call for further research. Biomed. Pharmacoter..

[8] Nascimento A.P.S., Duarte M.E.M., Rocha A.P.T., Barros A.N. (2025). Valorization of avocado (*Persea americana*) peel and seed: Functional potential for food and health applications. Antioxidants.

[9] Tremocoldi M.A., Rosalen P.L., Franchin M., Massarioli A.P., Denny C., Daiuto É.R., Paschoal J.A.R., Melo P.S., Alencar S.M.D. (2018). Exploration of avocado by-products as natural sources of bioactive compounds. PLoS ONE.

[10] Ferreira S.M., Falé Z., Santos L. (2022). Sustainability in skin care: Incorporation of avocado peel extracts in topical formulations. Molecules.

[11] Castromonte M., Wacyk J., Valenzuela C. (2020). Encapsulación de extractos antioxidantes desde sub-productos agroindustriales: Una revisión. Rev. Chil. Nutr..

[12] Noreen H., Semmar N., Farman M., McCullagh J.S. (2017). Measurement of total phenolic content and antioxidant activity of aerial parts of medicinal plant *Coronopus didymus*. Asian Pac. J. Trop. Med..

[13] Lyu X., Agar O.T., Barrow C.J., Dunshea F.R., Suleria H.A. (2023). Phenolic compounds profiling and their antioxidant capacity in the peel, pulp, and seed of Australian grown avocado. Antioxidants.

[14] Halliwell B. (2024). Understanding mechanisms of antioxidant action in health and disease. Nat. Rev. Mol. Cell Biol..

[15] Jia Z., Dumont M.J., Orsat V. (2016). Encapsulation of phenolic compounds present in plants using protein matrices. Food Biosci..

[16] Mohammadian M., Waly M.I., Moghadam M., Emam-Djomeh Z., Salami M., Moosavi-Movahedi A.A. (2020). Nanostructured food proteins as efficient systems for the encapsulation of bioactive compounds. Food Sci. Hum. Wellness.

[17] Galisteo-González F., Molina-Bolívar J.A. (2014). Systematic study on the preparation of BSA nanoparticles. Colloids Surf. B Biointerfaces.

[18] Cerda-Opazo P., Gotteland M., Oyarzun-Ampuero F.A., Garcia L. (2021). Design, development and evaluation of nanoemulsion containing avocado peel extract with anticancer potential: A novel biological active ingredient to enrich food. Food Hydrocoll..

[19] Sahyon H.A., El-Shafai N.M., Elnajjar N., Althobaiti F., Aldhahrani A., Alharbi N.S., Shoair A.G., El-Mehasseb I.M. (2023). Avocado peel extract loaded on chitosan nanoparticles alleviates urethane toxicity that causes lung cancer in a mouse model. Int. J. Biol. Macromol..

[20] Visentini F.F., Perez A.A., Santiago L.G. (2023). Bioactive compounds: Application of albumin nanocarriers as delivery systems. Crit. Rev. Food Sci. Nutr..

[21] Jahanshahi M., Najafpour G., Rahimnejad M. (2008). Applying the Taguchi method for optimized fabrication of bovine serum albumin (BSA) nanoparticles as drug delivery vehicles. Afr. J. Biotechnol..

[22] Lamichhane S., Lee S. (2020). Albumin nanoscience: Homing nanotechnology enabling targeted drug delivery and therapy. Arch. Pharm. Res..

[23] Xu X., Hu J., Xue H., Hu Y., Liu Y.N., Lin G., Liu L., Xu R.A. (2023). Applications of human and bovine serum albumins in biomedical engineering: A review. Int. J. Biol. Macromol..

[24] Pedrozo R.C., Antônio E., Khalil N.M., Mainardes R.M. (2020). Bovine serum albumin-based nanoparticles containing the flavonoid rutin produced by nano spray drying. Braz. J. Pharm. Sci..

[25] Khella K.F., Abd El Maksoud A.I., Hassan A., Abdel-Ghany S.E., Elsanhoty R.M., Aladhadh M.A., Abdel-Hakeem M.A. (2022). Carnosic acid encapsulated in albumin nanoparticles induces apoptosis in breast and colorectal cancer cells. Molecules.

[26] Martano S., Cascione M., Giotta L., Rizzello L., Di Corato R., Leporatti S., Rinaldi R., De Matteis V. (2025). Epigallocatechin-gallate loaded BSA nanoparticles as innovative anti-inflammatory agents in immature macrophages. Front. Bioeng. Biotechnol..

[27] Sánchez-Arreguin A., Carriles R., Ochoa-Alejo N., López M.G., Sánchez-Segura L. (2019). Generation of BSA-capsaicin nanoparticles and their hormesis effect on the *Rhodotorula mucilaginosa* yeast. Molecules.

[28] Carriles R., Zavala-García L.E., Nava-Coronel S., Sánchez-Arreguin A., López M.G., Sánchez-Segura L. (2021). Post-synthesis nanostructuration of BSA-Capsaicin nanoparticles generated by sucrose excipient. Sci. Rep..

[29] Bronze-Uhle E.S., Costa B.C., Ximenes V.F., Lisboa-Filho P.N. (2017). Synthetic nanoparticles of bovine serum albumin with entrapped salicylic acid. Nanotechnol. Sci. Appl..

[30] Rawel H.M., Czajka D., Rohn S., Kroll J. (2002). Interactions of different phenolic acids and flavonoids with soy proteins. Int. J. Biol. Macromol..

[31] Ozdal T., Capanoglu E., Altay F. (2013). A review on protein–phenolic interactions and associated changes. Food Res. Int..

[32] Liu J., Yong H., Yao X., Hu H., Yun D., Xiao L. (2019). Recent advances in phenolic–protein conjugates: Synthesis, characterization, biological activities and potential applications. RSC Adv..

[33] Sánchez-Segura L., Ochoa-Alejo N., Carriles R., Zavala-García L.E. (2018). Development of bovine serum albumin–capsaicin nanoparticles for biotechnological applications. Appl. Nanosci..

[34] Aboumanei M.H. (2026). Development of a Bovine Serum Albumin (BSA) Based Nanocarrier for Targeted Hepatic Delivery of Rosuvastatin: Preparation, Characterization, and Radiolabeling with Technetium-99 m for Biodistribution Studies. J. Pharm. Innov..

[35] Xiao Y., Ahmad T., Belwal T., Aadil R.M., Siddique M., Pang L., Xu Y. (2023). A review on protein based nanocarriers for polyphenols: Interaction and stabilization mechanisms. Food Innov. Adv..

[36] Bouwman A.M., Bosma J.C., Vonk P., Wesselingh J.H.A., Frijlink H.W. (2004). Which shape factor(s) best describe granules?. Powder Technol..

[37] Baldassarre F., Cacciola M., Ciccarella G. (2015). A predictive model of iron oxide nanoparticles flocculation tuning Z-potential in aqueous environment for biological application. J. Nanopart. Res..

[38] Khramtsov P., Kalashnikova T., Bochkova M., Kropaneva M., Timganova V., Zamorina S., Rayev M. (2021). Measuring the concentration of protein nanoparticles synthesized by desolvation method: Comparison of Bradford assay, BCA assay, hydrolysis/UV spectroscopy and gravimetric analysis. Int. J. Pharm..

[39] Patravale V.B., Date A.A., Kulkarni R.M. (2004). Nanosuspensions: A promising drug delivery strategy. J. Pharm. Pharmacol..

[40] Kumar B., Cumbal L. (2016). UV-Vis, FTIR and antioxidant study of *Persea americana* (Avocado) leaf and fruit: A comparison. Rev. Fac. Cienc. Quim..

[41] Yan Y., Zhu Q., Diao C., Wang J., Wu Z., Wang H. (2020). Enhanced physicochemical stability of lutein-enriched emulsions by polyphenol-protein-polysaccharide conjugates and fat-soluble antioxidant. Food Hydrocoll..

[42] Rawel H.M., Rohn S., Kruse H.P., Kroll J. (2002). Structural changes induced in bovine serum albumin by covalent attachment of chlorogenic acid. Food Chem..

[43] Hemlata-Gupta S., Tejavath K.K. (2021). ROS-mediated apoptosis induced by BSA nanospheres encapsulated with fruit extract of Cucumis prophetarum in various human cancer cell lines. ACS Omega.

[44] Cheng P.C., Pawley J.B. (2006). Interaction of light with botanical specimens. Handbook of Biological Confocal Microscopy.

[45] Tanjung Y.P., Dewi M.K., Gatera V.A., Barliana M.I., Joni I.M., Chaerunisaa A.Y. (2024). Factors affecting the synthesis of bovine serum albumin nanoparticles using the desolvation method. Nanotechnol. Sci. Appl..

[46] Sánchez-Segura L., Zaina S., Kú-González A.F., Guzmán-López J.A., Zavala-García L.E., López M.G. (2025). Effect of Crosslinking Using Heat on the Physicochemical Features of BSA–Capsaicin Nanoparticles. Pharmaceutics.

[47] Oppenheim R.C. (1981). Solid colloidal drug delivery systems: Nanoparticles. Int. J. Pharm..

[48] Langer K., Balthasar S., Vogel V., Dinauer N., von Briesen H., Schubert D. (2003). Optimization of the preparation process for human serum albumin (HSA) nanoparticles. Int. J. Pharm..

[49] Rahimnejad M., Najafpour G., Bakeri G. (2012). Investigation and modeling effective parameters influencing the size of BSA protein nanoparticles as colloidal carrier. Colloids Surf. A.

[50] Gough J.E., Scotchford C.A., Downes S. (2002). Cytotoxicity of glutaraldehyde crosslinked collagen/poly (vinyl alcohol) films is by the mechanism of apoptosis. J. Biomed. Mater. Res..

[51] Khan A.A., Paul A., Abbasi S., Prakash S. (2011). Mitotic and antiapoptotic effects of nanoparticles coencapsulating human VEGF and human angiopoietin-1 on vascular endothelial cells. Int. J. Nanomed..

[52] Liu L., Yu Y., Su K., Zhang X., Cheng W., He J. (2023). Polyphenol-bovine serum albumininteraction and its influence on protein structure. Trans. Chin. Soc. Agric. Eng..

[53] Skrt M., Benedik E., Podlipnik Č., Ulrih N.P. (2012). Interactions of different polyphenols with bovine serum albumin using fluorescence quenching and molecular docking. Food Chem..

[54] Elzoghby A.O., Samy W.M., Elgindy N.A. (2012). Albumin-based nanoparticles as potential controlled release drug delivery systems. J. Control. Release.

[55] Singleton V.L., Rossi J.A. (1965). Colorimetry of total phenolics with phosphomolybdic-phosphotungstic acid reagents. Am. J. Enol. Vitic..

[56] Fan H.Y., Nazari M., Raval G., Khan Z., Patel H., Heerklotz H. (2014). Utilizing zeta potential measurements to study the effective charge, membrane partitioning, and membrane permeation of the lipopeptide surfactin. Biochim. Biophys. Acta (BBA)-Biomembr..

[57] Syverud K., Chinga G., Johnsen P.O., Leirset I., Wiik K. (2007). Analysis of lint particles from full-scale printing trials. Appita J..

[58] Ferreira T., Rasband W. (2012). ImageJ User Guide. IJ 1.46r.

[59] Parakhonskiy B., Zyuzin M.V., Yashchenok A., Carregal-Romero S., Rejman J., Möhwald H., Wolfgang J.P., Skirtach A.G. (2015). The influence of the size and aspect ratio of anisotropic, porous CaCO_3_ particles on their uptake by cells. J. Nanobiotechnol..

